# Elevated Blood Lead Concentrations and Vitamin D Deficiency in Winter and Summer in Young Urban Children

**DOI:** 10.1289/ehp.9389

**Published:** 2006-12-18

**Authors:** Francis W. Kemp, Prasad V.S.V. Neti, Roger W. Howell, Peter Wenger, Donald B. Louria, John D. Bogden

**Affiliations:** 1 Department of Preventive Medicine and Community Health and; 2 Department of Radiology, University of Medicine and Dentistry of New Jersey–New Jersey Medical School, Newark, New Jersey, USA

**Keywords:** African-American, blood, children, Hispanic, lead, summer, vitamin D, winter

## Abstract

**Background:**

It is widely recognized that blood lead concentrations are higher in the summer than in winter. Although the effects of some environmental factors such as lead in dust on this phenomenon have been studied, relationships to sunlight-induced vitamin D synthesis have not been adequately investigated. Vitamin D status is influenced by the diet, sunlight exposure, age, skin pigmentation, and other factors, and may modify gastrointestinal lead absorption or release of lead stored in bones into the bloodstream.

**Objective and Methods:**

We collected paired blood samples from 142 young, urban African-American and Hispanic children in the winter and summer to study the seasonal increase in blood lead and its relationships to vitamin D nutrition, age, and race.

**Results:**

A winter/summer (W/S) increase in blood lead concentrations of 32.4% was found for children 1–3 years of age. There was a smaller W/S increase of 13.0% in children 4–8 years of age. None of the 51 Hispanic children had an elevated blood lead concentration (≥ 10 μg/dL) during the winter, and only one had an elevated summertime concentration. In contrast, elevated blood lead concentrations were frequent in the 91 African-American children, especially those 1–3 years of age. For the latter, the percentage with elevated blood lead levels increased from 12.2% in winter to 22.5% in summer. A 1.2% W/S increase in serum 25-hydroxy-vitamin D (serum 25-OH-D) concentrations was found for children 1–3 years of age. However, in children 4–8 years of age the W/S increase in serum 25-OH-D was much larger—33.6%. The percentages of children with low (< 16 μg/L) serum 25-OH-D concentrations were 12.0% in winter and 0.7% in summer and were consistently greater in African-American than in Hispanic children. The seasonal increases in blood lead and serum 25-OH-D in children 4–8 years of age were significantly associated.

**Conclusion:**

The higher summertime serum 25-OH-D concentrations for the 4- to 8-year-old children are likely caused by increased sunlight-induced vitamin D synthesis and may contribute to the seasonal increase in blood lead. Age and race are key factors that affect blood lead and vitamin D nutrition, as well as their interactions, in young urban children.

Most children with elevated blood lead concentrations and lead poisoning have been African-American or Hispanic children from low-income families who live in urban areas, especially in the northeastern and midwestern United States (Markowitz 2000; Mielke 1999; U.S. General Accounting Office 1999). Environmental lead concentrations are particularly high in cities in the Northeast, such as Newark, New Jersey, because of their history of high automobile traffic densities and the relatively old age of much of the housing. The inevitable lead exposure that results can cause lead poisoning, particularly in children 1–3 years of age. Their habit of putting their fingers in their mouths results in the ingestion of environmental lead, the primary route of lead exposure in young children (Manton et al. 2000; Markowitz 2000). The percentage of ingested lead absorbed is also greater in children than adults, and children’s low body weights increase lead’s toxicity (O’Flaherty 1998). Inhaled lead is also absorbed, and a portion of inhaled lead may be ingested as well (Markowitz 2000). Because ingestion of lead is the primary route of exposure in children, study of relationships between dietary nutrients and lead absorption and toxicity has been an active area of investigation [Bogden et al. 1997; Bruening et al. 1999; Centers for Disease Control and Prevention (CDC) 2002; Han et al. 2000; Schell et al. 2004].

People whose ancestors lived at higher latitudes usually have lighter skin than those whose ancestors lived closer to the equator. Darker skin provides a higher degree of protection against ultraviolet (UV) radiation damage to skin from sunlight exposure, but is less efficient than lighter skin for the sunlight-induced synthesis of vitamin D (Harris 2006; Moore et al. 2005). Thus, the evolution of race may be attributed partly to balancing the need to protect skin from excessive UV damage at lower latitudes with the need for adequate sunlight-induced vitamin D synthesis at higher latitudes. In support of this concept, recent results show that African-American women have lower serum 25 hydroxy-vitamin D (serum 25-OH-D) concentrations than Caucasian women living in the United States, and have an approximately 10-fold higher prevalence of vitamin D deficiency (42.4 vs. 4.2%) (Nesby-O’Dell et al. 2002). However, data are very limited on the vitamin D status of urban children. The need for additional research on vitamin D nutrition in children was emphasized at an October 2003 National Institutes of Health Conference titled “Vitamin D and Health in the 21st Century: Bone and Beyond” (Raiten and Picciano 2004).

An interesting facet of blood lead concentrations and lead poisoning is their seasonal variability. Specifically, blood lead concentrations and the prevalence of lead poisoning are higher in the warmer than in the colder months of the year (Baghurst et al. 1992; Laidlaw et al. 2005; Marrero et al. 1983; McCusker 1979; Yiin et al. 2000). In contrast to the United States, only limited seasonal variability was found in one study done in Australia (Gulson et al. 2000). The authors of the study suggested that the relatively small seasonal variability in Australia versus the United States can be attributed to smaller winter/summer (W/S) climate differences in Australia. The seasonal variability of blood lead in the United States is widely recognized, and some factors that may contribute to this phenomenon have been studied. Among factors that may explain the seasonal variability of blood lead levels are more outdoor play time of young children in contact with dust and soil, and more tracking into dwelling units of lead from major outdoor reservoirs such as urban soils during the summer (McCusker 1979; Yiin et al. 2000). Another possibility is that decreased precipitation may raise dust levels and thereby increase environmental lead exposure of children (Laidlaw et al. 2005).

Increased sunlight-induced vitamin D synthesis leading to increased gastrointestinal lead absorption or skeletal lead mobilization has also been hypothesized to be a cause of the increase in blood lead concentrations during the warmer months of the year (Moon 1994; Oliveira et al. 2002; Peraza et al. 1998), but has not been adequately studied. There is good evidence documenting the inhibition by lead of synthesis of 1,25 di-hydroxy-vitamin D (1,25-di-OH-D) in the kidney (Fullmer 1992, 1997), and circulating concentrations of this molecule are reduced in children with high blood lead concentrations (Box et al. 1981; Rosen et al. 1980). However, studies of the relationships between lead poisoning and serum 25-OH-D are few, were done more than two decades ago when blood lead concentrations of children were much higher, and have yielded inconsistent results (Rosen et al. 1980; Sorrell et al. 1977). No prior investigation has used paired W/S blood samples from the same children, and thus previous studies have limited value for assessing the role of vitamin D in the seasonal variation in blood lead.

The objectives of our present study were to use paired W/S blood samples to *a*) verify the summertime increase in blood lead in children at current levels of environmental lead exposure in a northeastern city with substantial W/S ambient temperature differences, *b*) estimate the prevalence of vitamin D deficiency during the winter and summer in African-American and Hispanic children enrolled in a Women, Infants, and Children (WIC) program, and *c*) determine relationships between blood lead and serum 25-OH-D concentrations and the influence of season, age, and race on these relationships.

## Methods

### Subjects and blood sampling

To study relationships among blood lead levels, race and ethnicity, season of the year, and serum 25-OH-D concentrations in Newark children, we enrolled 142 children at a single WIC program site on our campus in Newark, New Jersey. They were consecutive children seen at the WIC center who were in the target age range of 1–8 years old, whose parents or guardians consented to their participation in the study, and who were willing to comply with the study protocol of providing two venous blood samples 6–7 months apart. All subjects were enrolled during the planned winter enrollment period of December 2001 through March 2002, and returned for the planned summertime follow-up during July, August, or September 2002.

We administered a short questionnaire to obtain information on subject demographic and other data, including child age, self-reported race/ethnicity (African-American or Hispanic white), family income, and home address. Whole blood was collected by venipuncture into certified trace-metal-free heparinized (no. 369735) and anticoagulant-free (no. 369618) Vacutainers (Becton-Dickinson, Rutherford, NJ). We obtained a second venous blood sample for all of the participating children about 6–7 months after the first sample during July–September 2002. All blood samples were collected by a single pediatric phlebotomist. The mean ± SE interval between collection of the winter and summer blood samples was 6.2 ± 0.1 months.

All participating parents or guardians provided informed consent after the study requirements and procedures were explained to them. The study protocol was approved by the UMDNJ/Newark Institutional Review Board. Modest stipends were given to parents to provide an incentive to participate and partial reimbursement for lost time at work and/or travel costs.

### Weather

National Weather Service data for Newark Liberty International Airport (latitude: 40° 44’ N; longitude: 74° 10’ W) were accessed to provide an estimate of daily ambient atmospheric temperatures to which subjects were exposed during the study period.

### Whole blood lead and serum 25-OH-D analyses

The whole blood samples collected were delivered from the WIC site to our laboratory within 4 hr of collection. Blood specimens were processed to separate aliquots of whole blood and serum on arrival in the laboratory. Trace-element-free Vacutainers containing the anticoagulated whole blood were mixed on a blood rocker for 2 min, and two aliquots of the mixed blood were transferred into prerinsed cryovials (no. 430658; Corning, Corning, NY) for storage at –70°C until analysis for lead. Serum was obtained by centrifugation of the anticoagulant-free vacutainer tubes at 1,000 × *g* for 10 min, and aliquots of serum were also transferred into prerinsed cryovials for storage at –70°C until analysis for serum 25-OH-D.

Our methods for blood lead analyses and quality control have been previously described (Sowers 2002a, 2002b). Briefly, whole blood lead concentrations of all samples were measured in triplicate with a model Z5100 atomic absorption spectrophotometer with an electrothermal heated graphite atomizer with Zeeman background correction (PerkinElmer Corp., Norwalk, CT) using a modification of the method of Miller et al. (1987). The replicate values were averaged. Each of two different quality control samples was routinely analyzed at the beginning and at the end of each analytical run. Some quality control specimens were obtained from Wadsworth Laboratories, State of New York Department of Health, Lot NYS048 (Albany, NY). In addition, we also analyzed one of several lots of Lyphochek Level 1 and Level 3 Human Whole Blood Controls obtained from Bio-Rad (Anaheim, CA). The mean difference in lead concentrations between the start and end of each analytical run was < 4.1% for the control specimens. The certified value for NYS048 was 5.9 ± 0.4 μg/dL; our analyses of 96 aliquots of this sample yielded a mean (± SD) concentration of 6.26 ± 0.42 μg/dL (coefficient of variation = 6.7%). Our laboratory analyses of the 3 Lyphochek Level 1 specimens and the one Lyphochek Level 3 specimen yielded mean lead concentrations of 8.92 ± 0.37 μg/dL (*n* = 45; Lyphochek acceptable range = 7.0–10.4 μg/dL), 5.99 ± 0.28 μg/dL, (*n* = 25; Lyphochek acceptable range = 4.0–6.0 μg/dL), 8.89 ± 0.83 μg/dL (*n* = 14; Lyphochek acceptable range = 6.6–9.0 μg/dL), and 50.80 ± 2.12 μg/dL (*n* = 2; Lyphochek acceptable range = 41.9–56.7 μg/dL). Thus, all mean values for the Lyphochek samples were within the acceptable ranges.

Serum concentrations of 25-OH-D are generally accepted as the best way to assess vitamin D status and the presence of deficiency (Hanley and Davison 2005; Holick 2003; Hollis 2005). We conducted assays for serum 25-OH-D on paired W/S samples of all 142 subjects enrolled. Serum 25-OH-D is considered to be stable when stored in the frozen state (DiaSorin 2003). Therefore, W/S sample pairs were always thawed and analyzed at the same time to provide more accurate values of W/S differences in concentration. Serum 25-OH-D concentrations were determined in duplicate and the mean was calculated for each sample. We used a commercially available radioimmunoassay obtained from DiaSorin Inc. (Stillwater, MN) for the determination of 25-OH-D_3_ (cholecalciferol) and 25-OH-D_2_ (ergocalciferol). The technique uses the competitive binding of the extracted forms of the serum 25-OH-D and a ^125^I 25-OH-D_3_ tracer with a 25-OH-D_3_ specific goat antiserum. Addition of donkey anti-goat serum forms a precipitating complex permitting the quantitative determination of the sum of the 25-OH-D_2_ and 25-OH-D_3_ concentrations. Two human serum controls provided by the manufacturer, representing low-normal and high-normal concentrations, were analyzed at the beginning and end of each set of analyses to assess the reproducibility and accuracy of the assay. The average within-run difference from the mean concentration for each control was 2.2%. For the 284 samples from children and the 32 control sample analyses, the coefficient of variation for sample replicates was 6.9%. Generally, the accuracy of the assay was very good, with concentrations for both controls falling within the acceptable ranges. In two instances in which control means were not within the acceptable range, an adjustment in the standard curve was made to correct for this observation.

### Statistical evaluation of data

We divided the children into two age categories, 1–3 and 4–8 years of age, for data analysis because blood lead concentrations and the incidence of lead poisoning are generally highest between 1 and 3 years of age, and because national dietary guidelines use exactly the same age ranges in setting nutrient intake standards (the Dietary Reference Intakes) for all essential macronutrients and micronutrients, including vitamin D (Food and Nutrition Board–Institute of Medicine 1997).

Descriptive statistics for blood lead and serum 25-OH-D are presented as the mean ± SE. We calculated Pearson correlation coefficients to evaluate the relationships between blood lead and serum 25-OH-D concentrations. The General Linear Models (GLM) procedure (SAS release 8.02; SAS Institute, Cary, NC) was used to provide a repeated measures model of winter and summer blood lead and serum 25-OH-D concentrations for children of different ages and races. Paired *t*-tests were also used to evaluate W/S differences for blood lead and serum 25-OH-D concentrations.

## Results

**Figure 1:**
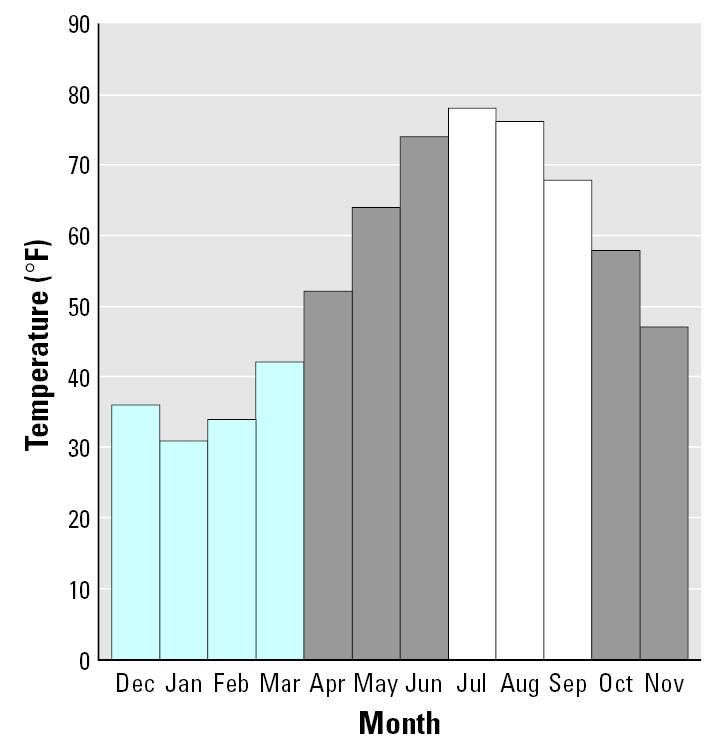
Mean monthly ambient atmospheric temperatures during the 12-month period of December 2001 to November 2002. Mean temperatures (° F) are from the National Weather Service for Newark (NJ) Liberty International Airport. Bars in blue are the months defined as “winter” and bars in white are the months defined as “summer.”

Figure 1 displays mean monthly ambient atmospheric temperatures for Newark, New Jersey, during the study period. The mean December–March temperature was 4.2°C (39.5°F) and the mean July–September temperature was 24.6°C (76.3°F), a W/S difference of 20.4°C (36.8°F). Table 1 summarizes the race/ethnicity of the 142 participating children.

**Figure 2:**
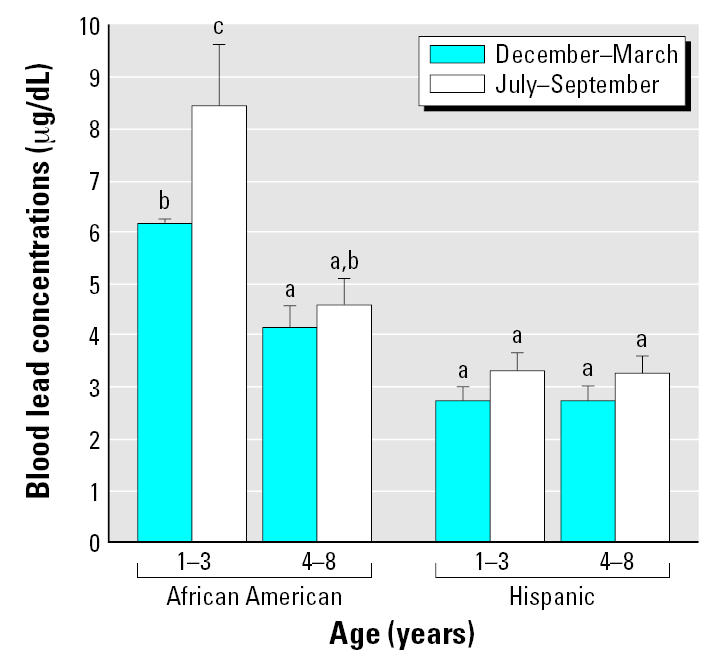
Mean blood lead concentrations of 142 children 1–8 years of age by age group, race, and season. Error bars depict SEs. Bars not marked by the same letter differ significantly (SAS-GLM, *p* < 0.05). Hispanic children: 1–3 years of age, *n* = 29; 4–8 years of age, *n* = 22. African-American children: 1–3 years of age, *n* = 49; and 4–8 years of age, *n* = 42.

Mean ± SE blood lead concentrations for the 78 1- to 3-year-old Hispanic and African-American children studied were 4.94 ± 0.45 μg/dL during the winter and 6.54 ± 0.82 μg/dL during the summer months, a significant (paired *t*-test, *p* = 0.0019) W/S increase of 32.4%. There was a significant (paired *t*-test, *p* = 0.0097) but smaller W/S increase of 13.0% in blood lead from 3.68 ± 0.31 to 4.16 ± 0.36 μg/dL for the 64 children 4–8 years of age. There was a significant association between the winter and summer blood lead concentrations (*r* = 0.851, *p* < 0.0001). Figure 2 shows that blood lead concentrations and the W/S difference in blood lead concentrations differed substantially among race and age subgroups of the children. The highest blood lead levels were found in the African-American children 1–3 years of age, whereas the lowest levels were found in the Hispanic children.

**Figure 3:**
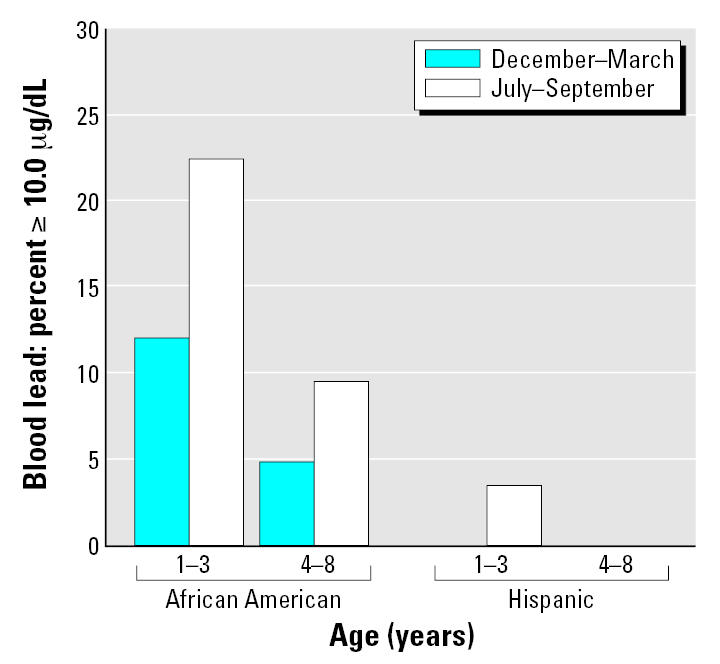
Percentages of children with blood lead concentrations ≥ 10.0 μg/dL by age group, race, and season. Hispanic children: 1–3 years of age, *n* = 29; 4–8 years of age, *n* = 22. African-American children: 1–3 years of age, *n* = 49; and 4–8 years of age, *n* = 42. No Hispanic child 1–3 years of age had an elevated blood lead concentration during the winter, but one had an elevated blood lead concentration during the summer. No Hispanic child 4–8 years of age had an elevated blood lead concentration during the winter or summer.

**Figure 4:**
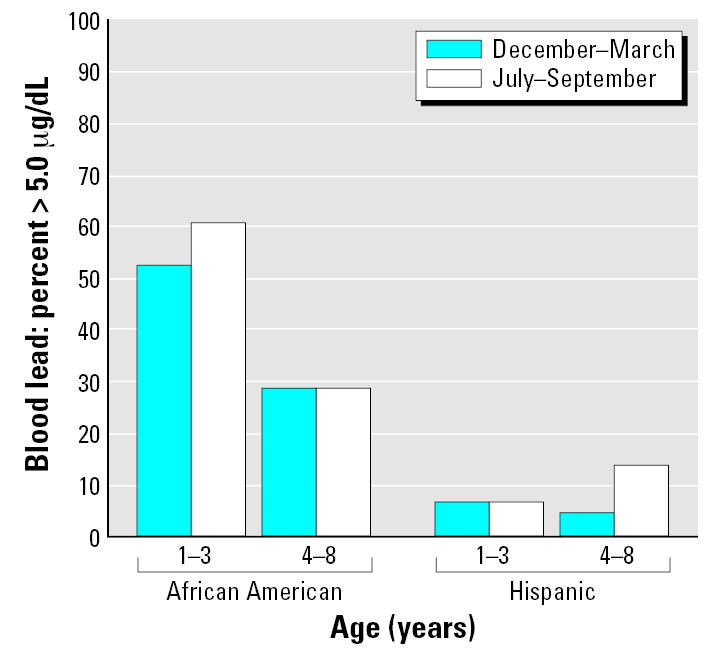
Percentages of children with blood lead concentrations > 5.0 μg/dL by age group, race, and season. Hispanic children: 1–3 years of age, *n* = 29; 4–8 years of age, *n* = 22. African-American children: 1–3 years of age, *n* = 49; and 4–8 years of age, *n* = 42.

Figure 3 depicts the percentages of the 142 children with elevated (≥ 10 μg/dL) blood lead concentrations. None of the 51 Hispanic children had an elevated blood lead concentration (≥ 10 μg/dL) during the winter, and only one had an elevated summertime concentration. In contrast, elevated blood lead concentrations were frequent in the 91 African-American children enrolled at the same WIC site, especially those children 1–3 years of age. For the latter, the percentage with elevated blood lead levels increased from 12.2% in winter to 22.5% in summer. Figure 4 depicts children with blood lead concentrations > 5 μg/dL; concentrations above this cutoff were also more frequent in African-American than in Hispanic children.

**Figure 5:**
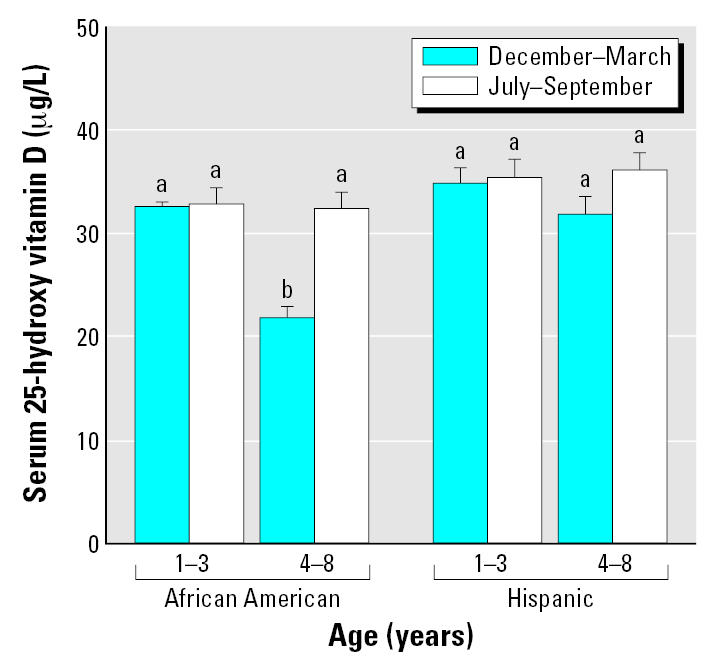
Mean serum 25-OH-D concentrations of 142 1- to 8-year-old children by age group, race, and season. Error bars depict SEs. Bars not marked by the same lower case letter differ significantly (SAS-GLM, *p* < 0.05). Hispanic children: 1–3 years of age, *n* = 29; 4–8 years of age, *n* = 22. African-American children: 1–3 years of age, *n* = 49; and 4–8 years of age, *n* = 42.

Children 1–3 years of age had serum 25-OH-D concentrations during the winter and summer of 33.4 ± 1.2 μg/L and 33.8 ± 1.1 μg/L, respectively, a small nonsignificant W/S increase of 1.2%. However, for children 4–8 years of age, serum 25-OH-D increased from 25.3 ± 1.2 to 33.8 ± 1.1 μg/L, a significant (paired *t*-test, *p* < 0.0001) increase of 33.6% that was much larger than the W/S increase found in the 1- to 3-year-old children. There was a significant association between the winter and summer serum 25-OH-D concentrations (*r* = 0.635, *p* < 0.0001). Figure 5 shows that serum 25-OH-D concentrations and the W/S difference in these concentrations differed among race and age subgroups of the children. The lowest wintertime concentrations and largest seasonal increase were found in the 4- to 8-year-old African-American children.

**Figure 6:**
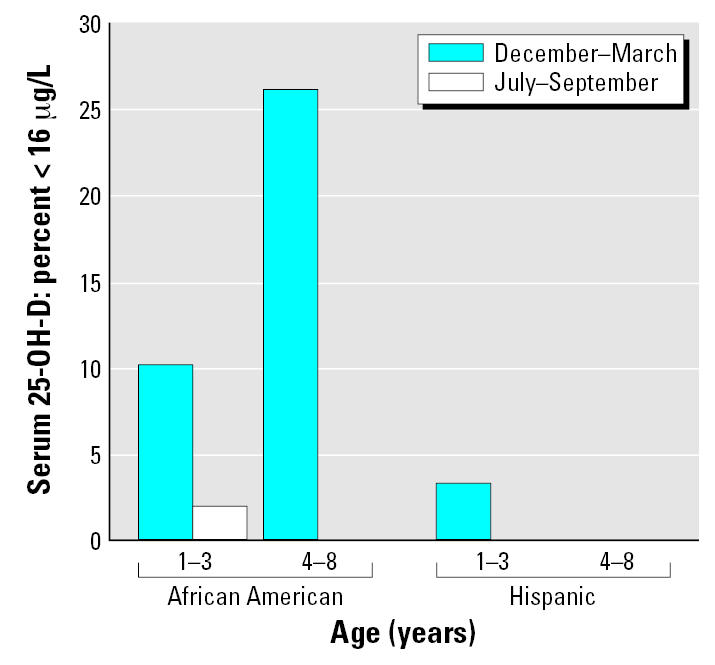
Percentages of children with serum 25-OH-D concentrations < 16 μg/L (40.0 nmole/L) by age group, race, and season. Hispanic children: 1–3 years of age, *n* = 29; 4–8 years of age, *n* = 22. African-American children: 1–3 years of age, *n* = 49; and 4–8 years of age, *n* = 42. No African-American child 4–8 years of age had a serum 25-OH-D level below 16 μg/L in the summer. No Hispanic child had a serum 25-OH-D level below 16 μg/L with the single exception of a 1- to 3-year-old child with a low wintertime concentration.

For all children, the percentages with low (< 16 μg/L) serum 25-OH-D concentrations were 12.0% in winter and 0.7% in summer. However, Figure 6 shows that the percentage of children with low serum 25-OH-D concentrations was consistently greater in African-American than in Hispanic children. In fact, except for one child with a low wintertime serum 25-OH-D concentration, no Hispanic child had a serum 25-OH-D level < 16 μg/L.

**Figure 7:**
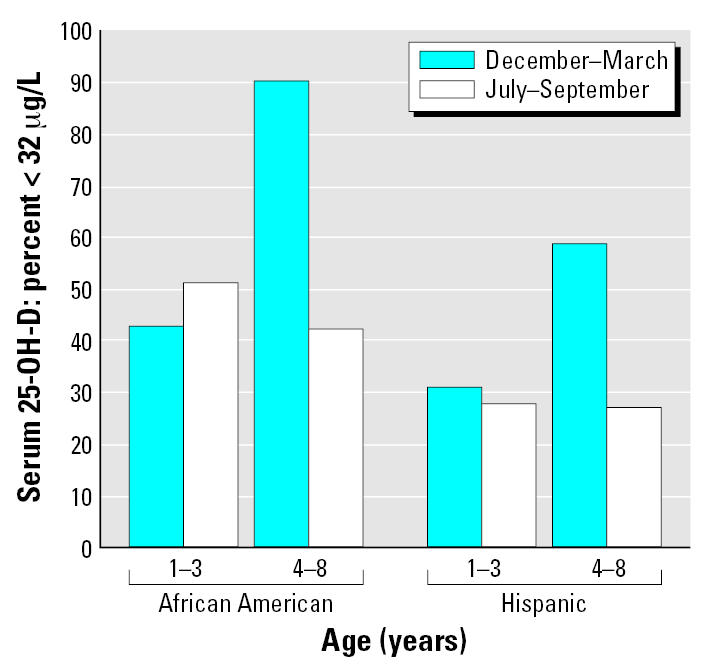
Percentages of children with serum 25-OH-D concentrations < 32 μg/L (80.0 nmole/L) by age group, race, and season. Hispanic children: 1–3 years of age, *n* = 29; 4–8 years of age, *n* = 22. African-American children: 1–3 years of age, *n* = 49; and 4–8 years of age, *n* = 42.

In Figure 6 we used a cutoff of 16 μg/L to define the lowest concentration of serum 25-OH-D that does not indicate deficiency. Various clinical laboratories in the United States typically consider that 12, 16, or 20 μg/L is the lower limit of their normal range, and thus lower values indicate vitamin D deficiency (Hanley and Davison 2005; Hollis 2005; Moore et al. 2005). The various laboratories may or may not use different cutoffs in the summer versus the winter. Using the above three cutoffs, the percentages of children with deficient levels were 0, 0.7, and 4.9% in the summer, but were considerably higher in the winter (3.5, 12.0, and 20.4%). These results document a moderate prevalence of vitamin D deficiency in this cohort of young minority-group Newark children. More recently, a higher cutoff of 32 μg/L for deficiency has been recommended by several investigators (Hanley and Davison 2005; Hollis 2005), and is now used as the lower limit of normal by at least one major clinical laboratory. Figure 7 shows that at this cutoff relatively high percentages of children (> 90% for 4- to 8-year-old African-American children in the winter) would be considered deficient.

**Figure 8:**
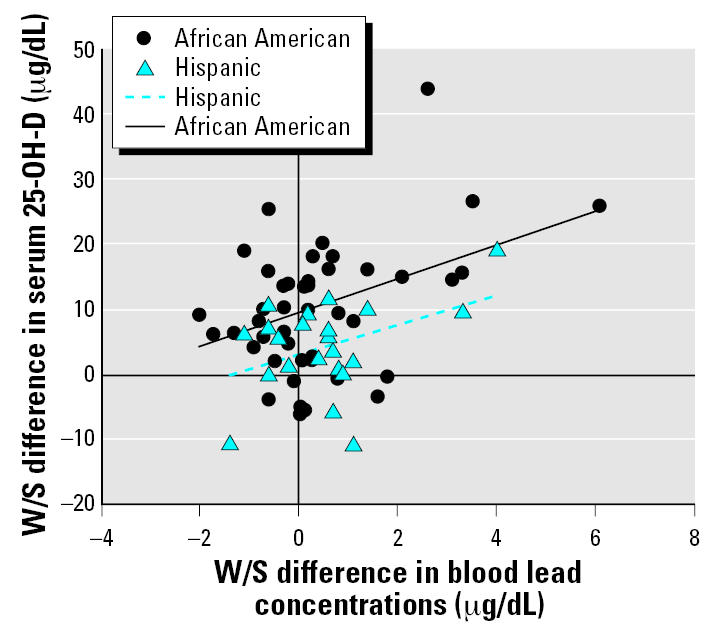
W/S difference in serum 25-OH-D versus W/S difference in blood lead concentration for 4- to 8-year-old African-American and Hispanic children. Hispanic children, *n* = 22; African-American children, *n* = 42. Values for Pearson correlation coefficients (*r*) for the Hispanic and African-American subgroups of children are 0.407 and 0.417, respectively.

The large seasonal increase in blood lead in the children 1–3 years of age was not accompanied by a significant increase in serum 25-OH-D concentrations. In contrast, the seasonal increase in blood lead for children 4–8 years of age was accompanied by a large increase in serum 25-OH-D levels, and the increases were significantly associated for all subjects (*r* = 0.383, *p* = 0.0018) and also for the subgroup of African-American children (*r* = 0.417, *p* = 0.0060) (Figure 8). For the Hispanic children the association was similar in magnitude (*r* = 0.407, *p* = 0.0599) but just beyond the level of *p* < 0.05 that we used to define statistical significance, due to the smaller number of Hispanic subjects studied.

## Discussion

### Lead poisoning and vitamin D deficiency

The results of the present study provide evidence for lead poisoning and vitamin D deficiency in the African-American children studied, whereas these diseases were virtually absent in their Hispanic counterparts. Elevated blood lead concentrations were most frequent in 1-to 3-year-old African-American children in the summer; in contrast, vitamin D deficiency was most frequent in 4- to 8-year-old African-American children during the winter.

The results replicate prior reports of summertime increases in blood lead (Baghurst et al. 1992; Laidlaw et al. 2005; Marrero et al. 1983; McCusker 1979; Yiin et al. 2000). Though the percentage increases were relatively large, more than 30% in children 1–3 years of age, the magnitude of the mean increase was relatively small, about 1.6 μg/dL for the same children. However, increases of this magnitude have been associated with reductions in IQ in young children even when the maximum blood lead concentration observed was < 10 μg/dL (Canfield et al. 2003; Lanphear et al. 2000), and thus are an important public health problem. Further, because many urban children have blood lead concentrations just below 10 μg/dL, the seasonal increase in the summer may be sufficient to increase their concentrations to ≥ 10 μg/dL for many children, as was the case for about 10% of the African-American children 1–3 years of age and 5% of the African-American children 4–8 years of age in the present study.

Because the seasonal increase of 32.4% in blood lead in the 1- to 3-year-old children was not accompanied by a significant increase in serum 25-OH-D concentrations, there must be other as yet unknown causes for this phenomenon in the children studied besides sunlight-induced vitamin D synthesis. These causes may include increased summertime exposure of children to lead in dust and soil, as has been reported in other studies (Laidlaw et al. 2005; McCusker 1979; Yiin et al. 2000). In contrast, the seasonal increase in blood lead of 13.0% in the 4- to 8-year-old children was accompanied by a large (33.6%) increase in serum 25 OH-D levels, and the increases were significantly associated (*r* = 0.383, *p* = 0.0018). Because vitamin D increases the gastrointestinal absorption of lead in experimental animals (Fullmer 1992; Moon 1994; Peraza et al. 1998), this association may be a causal relationship. Thus, in this age range, sunlight-induced vitamin D synthesis may be one of the causes of the summertime increase in blood lead.

The fact that not a single one of the 142 children had an undetectable blood lead concentration at either of the two blood collections documents the current relatively high level of environmental lead exposure of young Newark children.

These data display the expected seasonal increase in serum 25-OH-D for children 4–8 years of age but not those 1–3 years of age. Higher winter concentrations and/or relative lack of summertime sun exposure may explain the very small increase in the children 1–3 years of age versus the much larger increase in the children 4–8 years of age. The younger children may have higher and more consistent dietary intakes of vitamin D throughout the year that are caused by greater consumption of dairy products. The higher summertime (vs. winter) serum 25-OH-D concentrations for the 4- to 8-year-old children are likely attributed to increased sunlight-induced vitamin D synthesis and may contribute to the seasonal increase in blood lead. The strong positive associations between the winter and summer concentrations of blood lead (*r* = 0.851) and serum 25-OH-D (*r* = 0.635) demonstrate that those children with higher values of these concentrations in the winter also tend to have higher values in the summer.

Gulson et al. (2000), who conducted a study in Sydney, Australia, did not find a summertime increase in blood lead concentrations; this may be caused by the smaller W/S difference in mean outdoor temperatures of 9°C that they reported for Sydney, versus the 20.4°C difference in Newark for the months during which the present study was conducted. As in the Australian study, we used ambient temperature as a surrogate for hours of sunlight or sunlight intensity because all three of these variables are highly correlated.

The conversion in the kidney of 25-OH-D to 1,25-di-OH-D—the active form of vitamin D that is responsible for the intestinal absorption of calcium and, by analogy, lead—is tightly controlled by cellular vitamin D receptors and the vitamin D endocrine system. Thus, an argument could be made that the seasonal increase in serum 25-OH-D within physiologic (nondeficient) levels is not a cause of the seasonal increase in blood lead concentrations. However, the applicability of this argument to the present study is compromised by the following observations: *a*) 12% of the children we studied had serum 25-OH-D indicative of deficiency and thus did not have concentrations in the “physiologic” range; and *b*) experimental studies in the rat (Bogden et al. 1992) and chick (Fullmer 1992, 1997) have demonstrated that relationships among lead, dietary calcium, and 1,25-di-OH-D are complex and in some cases counterintuitive and therefore not predictable based solely on theoretical considerations. Thus, it is possible that the summertime increase in serum 25-OH-D is a cause of the seasonal increase in blood lead concentrations.

### Effects of race and ethnicity

Our results document the frequent occurrence of lead poisoning and vitamin D deficiency in the African-American children studied versus the virtual absence of both of these diseases in the Hispanic children studied to date. This is especially intriguing given that all of the children were Newark residents and members of WIC-eligible families with low incomes, and were enrolled at the same WIC site. Possible reasons for the lower blood lead concentrations in the Hispanic children include differences in housing environments, home maintenance and housekeeping practices that reduce lead exposure, indoor and outdoor activities that result in less lead exposure, and/or dietary differences that cause reduced absorption of ingested lead in the Hispanic children. However, additional research will be needed to identify the factors that are responsible for the lower blood lead levels in the Hispanic children than in the African-American children.

The data on blood lead are consistent with a population-based study of statistical associations done in Massachusetts that reported that African-American race but not Hispanic ethnicity was associated with a higher community case identification rate of lead poisoning in children (Sargent et al. 1995), and with a 1970–1976 study that found higher blood lead levels in African-American than in Hispanic children living in New York City (Billick et al. 1979).

Factors that may explain the differences in prevalence of vitamin D deficiency between the African-American and Hispanic children whom we studied include diet, time spent outdoors, quantity and intensity of sunlight exposure, clothing worn while outdoors, skin pigmentation, and use of sunscreens. Differences in the prevalence of obesity in the African-American and Hispanic children studied may also be a factor, because a higher body mass index in adults has been associated with lower serum 25-OH-D concentrations (Wortsman et al. 2000). However, additional study is required to provide insight into the relatively low prevalence of lead poisoning and vitamin D deficiency in the Hispanic compared to the African-American children studied.

Our results are consistent with prior studies that show a higher prevalence of vitamin D deficiency in African-American adolescents and adults than in Hispanic and other white subjects (Gordon et al. 2004; Harris 2006; Moore et al. 2005; Nesby-O’Dell et al. 2002), and suggest that deficiency begins at a relatively young age and may persist into adolescence and adulthood. They are also consistent with a recent study showing lower serum 25-OH-D concentrations in 4- to 8-year-old African-American than in white girls living in the Athens, Georgia, region of the United States (Stein et al. 2006). However, vitamin D deficiency was more frequent in the Newark children 4–8 years of age whom we studied than in the 168 African-American and white girls living in Georgia; this is likely caused at least partly by the lower latitude (about 34° N latitude for Athens, GA, versus 41° N latitude for Newark, NJ) and thus greater sunlight intensity and longer duration of hot weather in Georgia.

### Limitations of the study

One limitation of the present study is that the significant positive associations we found between the summertime increases in blood lead and serum 25-OH-D may not represent a causal relationship. Another limitation is that the prevalence of vitamin D deficiency and/or lead poisoning in children from urban families who are not WIC participants may differ from those of the WIC participants that we studied. The low family incomes needed to qualify for WIC eligibility are likely associated with living in housing that presents a greater risk for lead poisoning than that of Newark children from families with higher incomes. However, the regular nutrition advice about increasing dietary calcium and vitamin D intake and assistance with food purchasing provided to families at our WIC center may modify the risk of both lead poisoning (Bogden et al. 1997; CDC 2002) and vitamin D deficiency.

### Prevention of disease

We found a significant association between the summertime increases in blood lead and serum 25-OH-D concentrations for children 4–8 years of age, but not for children 1–3 years. In the children 4–8 years of age the seasonal increase in serum 25-OH-D explained about 17% of the variability in the corresponding increase in blood lead concentrations for both the African-American and Hispanic children. Thus, other factors are responsible for the remaining 83% of the variability in the summertime increase in blood lead concentrations. Furthermore, for the African-American children about 10% of those 1–3 years of age and 25% of those 4–8 years of age had low (< 16 μg/L) serum 25-OH-D concentrations during the winter. Therefore, limiting sun-exposure or dietary vitamin D for the sole purpose of reducing the summertime increase in blood lead levels, especially for children 4–8 years of age, does not seem prudent. However, it may be appropriate to discourage the use of vitamin D supplements by those children without vitamin D deficiency who are at-risk for lead poisoning until further research is conducted to determine if regular ingestion of the supplements increases blood lead concentrations.

It is well known that vitamin D deficiency can compromise skeletal development and bone quality and, if severe, cause rickets in children (Gartner and Greer 2003) or osteomalacia in adults (Meyer 2004). Further, recent evidence suggests that vitamin D deficiency is associated with increased risks of development of type 1 diabetes (Harris 2005; Hypponen et al. 2001), breast, colon, ovarian, and prostate cancers (Garland et al. 2006; Giovannucci 2005; Grau et al. 2003; Gross 2005), and multiple sclerosis (Munger et al. 2004). If further research demonstrates that vitamin D deficiency is a cause for one or more of these diseases, then efforts to identify and correct vitamin D deficiency in children may protect against the future development of one or more of these serious illnesses.

### Conclusion

To our knowledge, this is the first investigation to use paired winter and summer blood samples from the same children to study lead poisoning, vitamin D status, and their interactions. Thus, it provides this key advantage over the few prior studies of relationships between blood lead and vitamin D status (Box et al. 1981; Mahaffey et al. 1982; Rosen et al. 1980; Sorrell et al. 1977), all done > 25 years ago when blood lead concentrations of urban children were much higher. Prior studies also did not adequately consider the effects of age and race/ethnicity on relationships of vitamin D to the seasonal increase in blood lead, whereas the current study provides new evidence that shows that age and race/ethnicity are key variables that influence these relationships. In summary, lead poisoning and vitamin D deficiency are common in the young urban children we studied. In addition season of the year, age, and race/ethnicity appear to be key factors that influence blood lead concentrations and vitamin D nutrition, as well as their interactions, in children.

## Figures and Tables

**Table 1:** Participating children [no. (%)].

	Race/ethnicity		
Age (years)	Hispanic	African American	Totals
1–3	29 (20.4)	49 (34.5)	78 (54.9)
4–8	22 (15.5)	42 (29.6)	64 (45.1)
Totals	51 (35.9)	91 (64.1)	142 (100.0)

Of the Hispanic children, 30 were males and 21 were females. Of the African-American children, 45 were males and 46 were females.
